# Requirement of focal adhesion kinase in branching tubulogenesis

**DOI:** 10.1186/1423-0127-16-5

**Published:** 2009-01-12

**Authors:** Wei-Chun Wei, Anna K Kopec, Ming-Jer Tang

**Affiliations:** 1 Institute of Basic Medical Sciences, National Cheng Kung University Medical College, Tainan 701, Taiwan; 2 Department of Biochemistry & Molecular Biology, Michigan State University, East Lansing, MI 48824-1319 USA; 3 Department of Physiology, National Cheng Kung University Medical College, Tainan 701, Taiwan; 4 Center of Gene Regulation and Signal Transduction, National Cheng Kung University Medical College, Tainan 701, Taiwan

## Abstract

We previously demonstrated that α3β1 integrins are essential to hepatocyte growth factor (HGF)-independent branching tubulogenesis in Mardin-Darby Canine Kidney (MDCK) cells. However, the involvement of integrin downstream signaling molecules remains unclear. In the present study, we successfully isolated cell lines possessing different tubulogenic potentials from the MDCK cells; cyst clones (CA4, CA6) forming cystic structures when cultured in 0.3% type I collagen gel and mass clones (M610, M611, M612) forming aggregated masses. Cyst clones maintained cystic structure in 0.1% collagen gel, whereas mass clones spontaneously developed into tubules. Both clones exhibited various morphologies when cultured on a dish: cyst clones formed aggregated islands, while mass clones were more scattered and exhibited higher migration capacity. Among several focal adhesion machinery proteins examined, only the expression and phosphorylation level of focal adhesion kinase (FAK) in mass clones was higher than in cyst clones, while other proteins showed no obvious differences. However, overexpression of wild type FAK in CA6 cells did not facilitate branching tubule formation in 0.1% collagen gel. Targeted decrease in the expression level of FAK in M610 cells with the application of antisense cDNA resulted in a marked reduction of branching tubule formation in 0.1% collagen gel and showed a down-regulation of fibronectin assembly, which is known to promote tubulogenesis. In contrast, overexpression of wild type FAK in CA6 cells had no effect on fibronectin assembly. Taken together, our data demonstrates that FAK is required, but not sufficient for HGF-independent branching tubulogenesis in MDCK cells.

## Background

Epithelial branching tubulogenesis plays an essential role in the development of several tissues such as kidney, lung, salivary and mammary glands, and the study of the underlying mechanisms has been of both basic and clinical interest. Mardin-Darby canine kidney (MDCK) cells cultured in collagen gel are widely used as the simplest and most convenient model for studying tubulogenesis. Clone II 3B5, isolated from MDCK cells, can undergo morphogenesis and develop into polarized, elongated and branching tubule structures under the stimulation of hepatocyte growth factor (HGF) [1,2]. In our previous study, we successfully isolated several MDCK sub-clones, y224 and m634, that exhibited either cystic or tubular phenotypes in low concentration of collagen gel without HGF stimulation [3,4]. These cells became excellent models for studying novel regulatory mechanisms of branching tubulogenesis.

It has been demonstrated that extracellular matrix proteins (ECM) are key regulators for branching morphogenesis. Fibronectin, for example, is synthesized by groups of adherent cells that assemble it into a fibrillar network [5,6]. Fibronectin is known to provide survival cues for MDCK cell morphogenesis and development of mouse submandibular salivary gland, kidney as well as lung [7-9]. Laminin, another example of ECM, is crucial in the end bud persistence and ductal elongation in the developing mammary gland [10]. Despite the importance of extracellular matrix proteins, integrins (the membrane receptors of ECM) are also of particular significance, as the potential of branching tubulogenesis in MDCK cells was inhibited by the decrease in α3 integrin expression [3]. Monoclonal antibodies against either α6 or β1 subunit were shown to reduce kidney tubulogenesis *in vitro *[11], while temporal and spatial changes in integrin expression promoted branching morphogenesis of the developing collecting system *in vivo *[12].

Focal adhesion proteins are downstream of ECM and interact with integrins. Among all focal adhesion complex proteins, focal adhesion kinase (FAK) is of greatest significance. FAK connects both integrin and growth factor receptors with the intracellular signaling pathways and has five tyrosine phosphorylation sites (Y397, Y407, Y577, Y861, Y925), each mediating a different cellular function. In general, FAK-mediated signaling affects cell motility, cell survival, and cell cycle progression [13,14]. Because of the FAK involvement in the downstream cascade of integrin signaling, we wished to examine the role FAK in renal branching tubulogenesis.

In the present study we isolated several clones from MDCK cell line, i.e. cyst and mass clones, according to the methods described previously [3]. When both clones were cultured in 0.1% collagen gel, cyst clones formed cysts, while mass cells formed tubular structures. The investigation of the protein profiles showed that FAK expression was higher in mass clones then cyst clones. Overexpression of wild type FAK in cyst clones did not facilitate branching tube morphology when cultured in collagen gel. However, decrease of FAK expression levels by antisense cDNA in mass clones resulted in a marked reduction in branching tubule morphology in collagen gel. Our data suggest that FAK is required, but not sufficient for renal branching tubulogenesis.

## Materials and methods

### Cell lines and transfection

Cells were maintained in Dulbecco's modified Eagle's medium (DMEM, GIBCO BRL) supplemented with 5% fetal bovine serum under 5% CO_2 _at 37°C. MDCK II 3B5 cells overexpressing HA epitope-tagged wild type FAK, FRNK were the gifts from Dr. Hong-Chen Chen (National Chung Hsing University, Taiwan). To generate cells stably expressing wild type FAK or antisense FAK cDNA, cells were grown on 60 mm dishes and transfected essentially using LipofectAMINE according to the manufacturer's protocol. Clones were selected in hygromycin-containing medium and screened by immunoblotting with anti-HA or anti-FAK.

### Materials

Fetal bovine serum and LipofectAMINE were purchased from Life Technologies, Inc. G418 sulfate and hygromycin were purchased from Calbiochem (San Diego, CA). Recombinant human HGF were purchased from Sigma (St. Louis, MO).

### Preparation of collagen gel

Type I collagen was prepared from rat tail tendons according to the established procedure [15]. The collagen stock solution (1%), was composed of 1 g (dry weight) of rat tail tendons dissolved in 100 ml 0.025 N acetic acid. For the preparation of 0.1% collagen gel, 1 vol of collagen stock was mixed with 5.7× DMEM (1 vol), 2.5% NaHCO_3 _(0.5 vol), 0.1 M HEPES (1 vol), 0.17 M CaCl_2 _(0.03 vol), 1N NaOH (0.03 vol), and 1× culture medium (6.34 vol) under chilled conditions.

### Tubulogenesis

For tubulogenesis assays, MDCK cells were suspended at a concentration of 2 × 10^4 ^cells/ml in cold collagen solution as described above. An aliquot (1 ml) of cell suspension was dispensed into a well of 6-well plate and allowed to gel for about 20 min at 37°C before adding 1 ml of medium containing 5% serum with or without HGF. The medium was changed daily. After 5 days, the tubules were photographed using a phase contrast microscope.

### Collection of cell lysate and Western blot

The cells were cultured on dish for 24 hrs. Cell lysates were harvested in modified RIPA buffer (150 mM NaCl, 1 mM EGTA, 50 mM Tris pH 7.4, 10% glycerol, 1% Triton X-100, 1% sodium deoxycholate, 0.1% SDS, and Complete™) and the lysates were analyzed by Western blot using antibodies against FAK, fibronectin, vinculin, talin, paxillin and Src (Transduction Lab), phosphorylated Y397, Y407, Y577, Y861, Y925 (Biosource), α2, α3, β1 integrin (Chemicon), HA (Roche), p130cas, β-actin (Sigma) and visualized using enhanced chemiluminescence (ECL) system (Amersham-Pharmacia).

### Migration assay

Cells were collected by trypsinization and suspended in serum-free medium at 5 × 10^5 ^cells/ml. Migration assays were carried out in a Neuro Probe 48-well chemotaxis chamber (Cabin John, MD). The medium containing collagen (10 μg/ml) was added to the lower chamber. The lower and upper chambers were separated by a polycarbonate membrane (8 μm pore size, Poretics, Livermore, CA). Cells were allowed to migrate for 6 hrs at 37°C in a humidified atmosphere containing 5% CO_2_. The membrane was fixed in methanol for 10 min and stained with modified Giemsa stain (Sigma) for 1 hr. Cells on the upper side of the membrane were removed by cotton swabs. Cells on the lower side of the membrane were counted using a light microscope at 200× magnification.

### Plasmid construction

Total RNA was isolated from cultured MDCK cells by the standard guanidium isothiocyanate extraction method. Reverse transcription (RT) and polymerase chain reaction (PCR) were performed. Primers used to amplify a 712 bp segment of canine FAK cDNA were as follows: forward primer 5'-TACCTACCGAGGTCTCAGTTAG-3' (nucleotides 1305 to 1326 in human FAK cDNA) and reverse primer 5'-GCGCTGATCTTCTTCCATTTCC-3' (nucleotides 1995 to 2016 in human FAK cDNA). This segment is conserved in human, rat, mouse, and chicken FAK. The 712 bp PCR product was further mixed with XhoI cutting sequence, purified from 1% agarose, cleaved with XhoI endonuclease, and ligated into pMSCVhyg to create pMSCVFAKhyg, which was finally transfected into *E. coli*. The canine FAK sequence is approximately 89% homologous with the human FAK cDNA.

### Fibronectin assembly assay

The method to determine the fibronectin matrix assembly [16] was modified. To detect the amount of assembled fibronectin in cells cultured on dish, MDCK cells were cultured in the culture medium for 24 hrs then rinsed twice with ice cold PBS. Cells were lysed in DOC lysis buffer (2% sodium deoxycholate, 20 mM Tris-Cl, pH 8.8, 2 mM phenylmethysulfonyl fluoride, 2 mM EDTA, 2 mM iodoacetic acid, and 2 mM N-ethylmaleimide) and cell lysate was harvested using a cell scraper (Nunc). To detect the amount of assembled fibronectin MDCK cells were cultured in 0.1% collagen gel for 6 days. Collagen gel was digested by 0.2% collagenase under 37°C for 10 min. Cells were collected by centrifugation at 1200 rpm and then lysed in the DOC lysis buffer. Lysates were centrifuge for 15 min at 13,000 rpm in 4°C to separate DOC-insoluble matrix. The DOC-insoluble fibronectin was then dissolved in SDS-containing buffer (1% SDS, 20 mM Tris-Cl, pH 8.8, 2 mM PMSF, 2 mM EDTA, 2 mM iodoacetic acid, 2 mM N-ethylmaleimide) and analyzed by Western blot to detect fibronectin.

## Results

**Figure 1:**
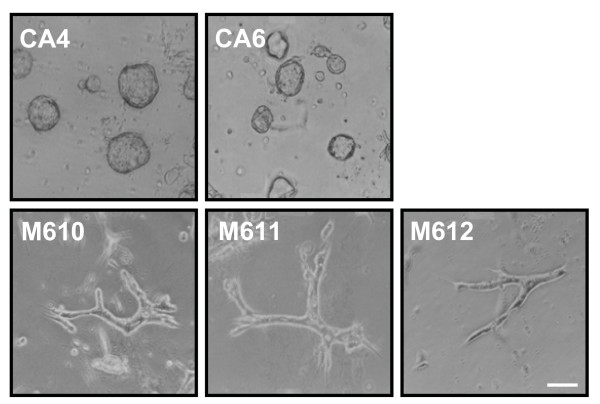
**Morphogenesis of cyst and mass clones cultured in 0.1% type I collagen gel**. Cyst clones (CA4, CA6) and mass clones (M610, M611, M612) were cultured in 0.1% collagen gel for 6 days. Images were taken under a light microscope. Bar = 100 μm.

MDCK cells form two different phenotypes when cultured in 0.3% type I collagen gel. Most of these cells develop into polarized cysts with an obvious central cavity, while the others form cell aggregates [3]. In the present study, we successfully isolated MDCK subclones that consistently developed either cysts (cyst clones – CA4, CA6) or cell aggregates (mass clones – M610, M611, M612) in 0.3% collagen gel for at least five passages. When these clones were cultured in 0.1% collagen gel for 6 days, cyst clones continued to display cystic morphogenesis, whereas mass clones developed into tubular structures (Fig. 1).

**Figure 2:**
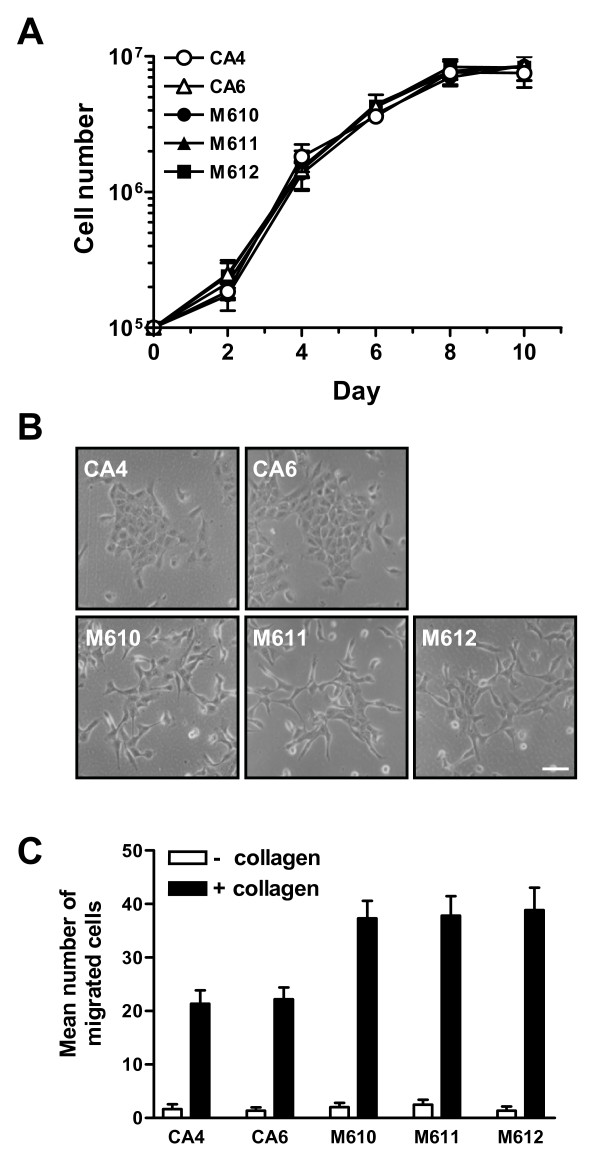
**Characteristics of cyst and mass clones cultured on dish**. **(a) **Morphology of cyst clones (CA4, CA6) and mass clones (M610, M611, M612) cultured on dish for 24 hrs. Images were taken under a light microscope. Bar = 30 μm. **(b) **Growth curve of cyst and mass clones cultured on dish for 10 days. **(c) **Cyst clones and mass clones subjected to migration assays using collagen as a chemoattractant. Migrated cells were fixed, stained, and counted under a light microscope. Values (mean ± S.E.) are from 15 fields and 3 independent experiments.

Although cyst clones and mass clones exhibited different morphogenesis in 0.1% collagen gel, they showed similar growth capability (Fig. 2a). A notable difference in the morphology between cyst clones and mass clones was observed when the cells were cultured on the culture dish. Cyst clones revealed a well-developed association between neighboring cells and formed aggregated islands. In contrast, mass clones showed more scattered morphology when cultured on the dish (Fig. 2b). The scattered morphology indicates that mass clones might have greater motility than cyst clones. To test this hypothesis, we used Boyden chamber transwell assay to analyze the migration ability of these two clones. Under collagen stimulation, mass clones showed higher migration ability than cyst clones (Fig. 2c).

**Figure 3:**
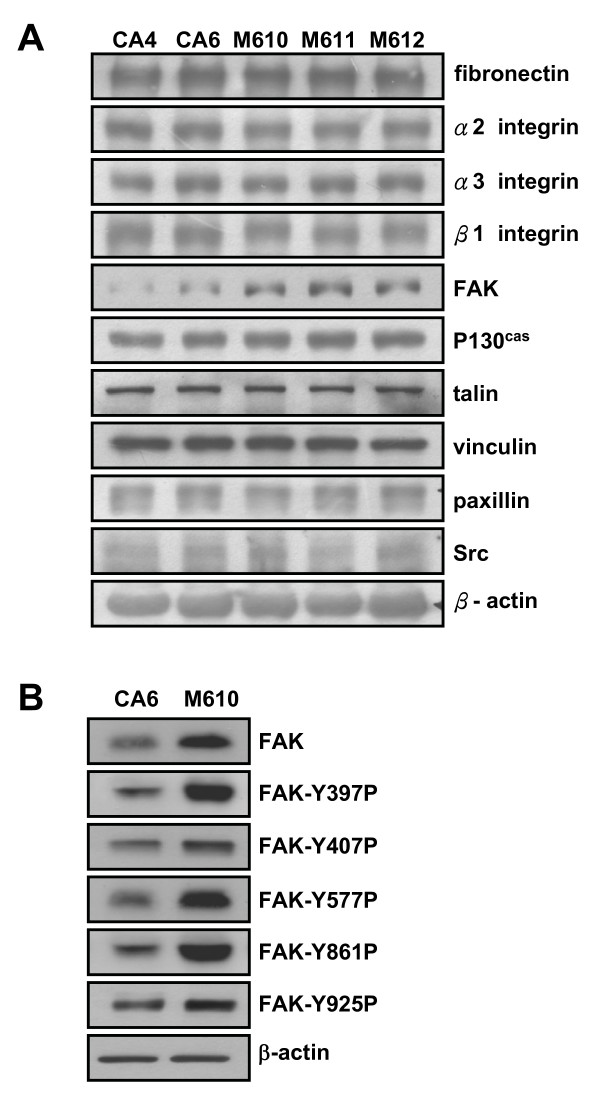
**Focal adhesion proteins expression in cyst and mass clones**. **(a) **Cyst clones (CA4, CA6) and mass clones (M610, M611, M612) were cultured on dish for 24 hrs. Lysates were analyzed by Western blot using antibodies to detect fibronectin, α2, α3, β1 integrin, FAK, P130cas, talin, vinculin, paxillin, Src, and β-actin. **(b) **CA6 and M610 cells were cultured on dish for 24 hrs. Lysates were analyzed by Western blot using antibodies to detect FAK and Y397, Y406, Y577, Y861, Y925 phosphorylated FAK levels.

Since migration ability is highly regulated by focal adhesion related proteins, we reasoned that the expression of these proteins may be involved in regulating cell morphogenesis. We analyzed some focal adhesion related proteins including extracellular matrix (fibronectin), receptors for extracellular matrix (α2, α3 and β1 integrins) and focal adhesion complex proteins (FAK, p130cas, talin, vinculin, paxillin, Src). Among these proteins, none of them showed apparent differences between these two clones except for the expression levels of FAK, which was markedly higher in mass than cyst clones (Fig. 3a). We furthered detected the phosphorylation level of different tyrosine phosphorylation sites on FAK in CA6 and M610 cells. As shown in Fig. 3b, M610 cells showed higher phosphorylation level in all FAK tyrosine phosphorylation sites compared to CA6 cells. These data suggest that FAK may be involved in regulating tubule formation.

**Figure 4:**
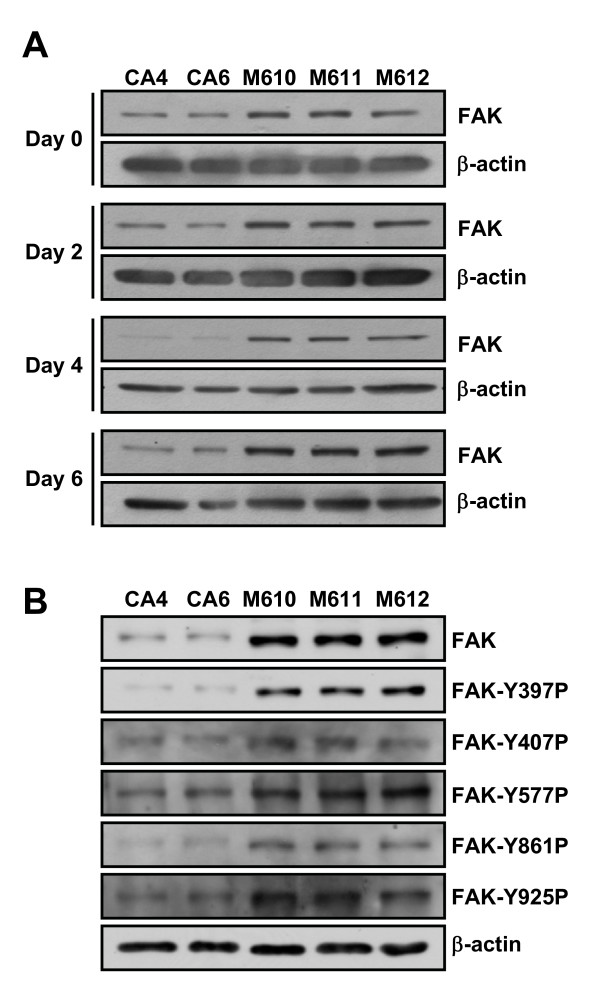
**Comparison of FAK and FAK phosphorylation levels between cyst and mass clones cultured in 0.1% collagen gel**. **(a) **Cyst clones (CA4, CA6) and mass clones (M610, M611, M612) were cultured in 0.1% collagen gel for indicated time. Lysates were analyzed by Western blot using antibodies to detect FAK and β-actin. **(b) **Cyst clones (CA4, CA6) and mass clones (M610, M611, M612) were cultured in 0.1% collagen gel for 6 days. Lysates were analyzed by Western blot using antibodies to detect FAK and Y397, Y406, Y577, Y861, Y925 phosphorylated FAK.

We further investigated whether FAK expression and phosphorylation level is higher in mass than in cyst clones when cultured in 0.1% collagen gel. Cyst and mass clones were cultured in 0.1% collagen gel for 6 days and FAK expression and phosphorylation levels were examined. When cells were cultured in 0.1% collagen gel, mass clones showed consistently higher FAK levels than cyst clones within 6 days (Fig. 4a). Furthermore, M610 cells showed higher phosphorylation levels in all FAK tyrosine phosphorylation sites compared to CA6 cells when cultured in 0.1% collagen gel for 6 days (Fig. 4b).

**Figure 5:**
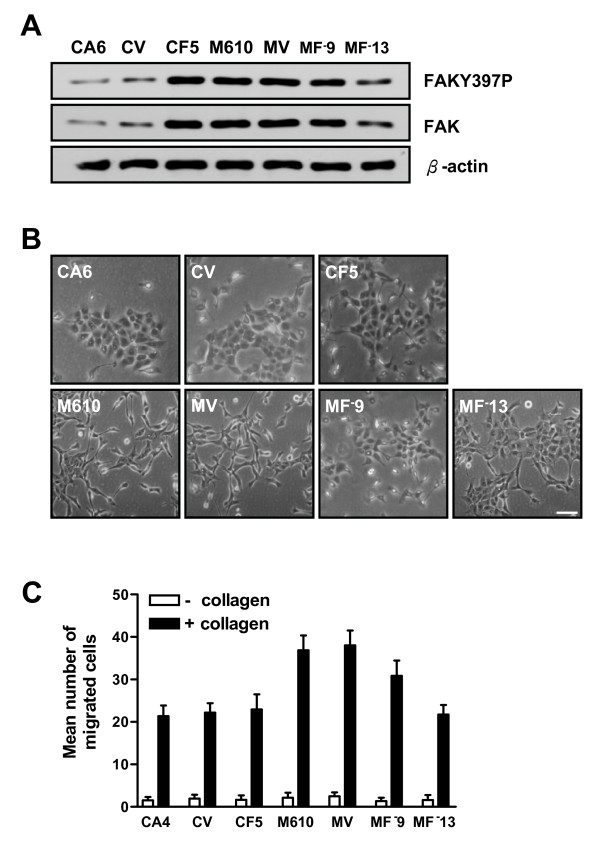
**Morphology and migration ability of FAK transfectants in cyst and mass clones**. **(a-b) **CA6, M610, control clones (CV, MV), wild type FAK overexpressed-CA6 cell (CF5), or antisense FAK overexpressed-M610 cells (MF^-^9, MF^-^13) were cultured on dish for 24 hrs. **(a) **Lysates were analyzed by Western blot using antibodies to detect FAK and β-actin. **(b) **Cell morphology; images were taken under a light microscope. Bar = 30 μm. **(c) **Cells were subjected to migration assays using collagen as a chemoattractant. Migrated cells were fixed, stained, and counted under a light microscope. Values (mean ± S.E.) are from 15 fields and 3 independent experiments.

In order to define the role of FAK in regulating tubule formation, we established several FAK transfectants in cyst and mass clones. To elevate the FAK expression level, wild type FAK was overexpressed in CA6 cells. In addition, antisense cDNA was used to decrease the FAK expression level in M610 cells. Elevation of FAK in CA6 cells increased FAK Y397 phosphorylation level. Meanwhile, reduction of FAK expression in M610 cells also diminished the FAK Y397 phosphorylation level (Fig. 5a). We further observed the morphology of those FAK transfectants when cultured on dish. FAK-overexpressed CA6 cells (CF5) formed aggregated islands similar to CA6 and control cells (CV). However, when antisense FAK-overexpressed M610 cells (MF^-^9, MF^-^13) were cultured on dish, they showed markedly reduced scattered morphology and elevated cell-to-cell association compared to M610 and control cells (MV) (Fig. 5b). To measure whether cell motility was altered by different FAK expression, migration capability of different FAK transfectants was analyzed by Boyden chamber with collagen as a chemoattractant. Increasing the expression level of FAK in CA6 cells did not elevate migration ability significantly; however decreasing the expression level of FAK in M610 cells reduced the migration ability in a dose-dependent manner (Fig. 5c).

**Figure 6:**
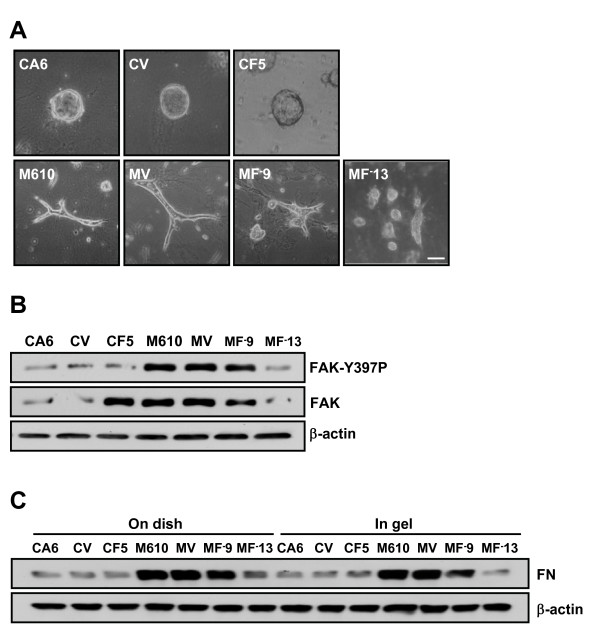
**Characteristics of FAK transfectants in cyst and mass clones cultured in 0.1% collagen gel**. **(a-b) **CA6, M610, control clones (CV, MV), wild type FAK overexpressed-CA6 cell (CF5), or antisense FAK overexpressed-M610 cells (MF^-^9, MF^-^13) were cultured in 0.1% collagen gel for 6 days. **(a) **Morphology of cells, images were taken under a light microscope. Bar = 100 μm. **(b) **Lysates were analyzed by Western blot using antibodies to detect FAK, Y397 phosphorylated FAK and β-actin. **(c) **CA6, M610, control clones (CV, MV), wild type FAK overexpressed-CA6 cell (CF5), or antisense FAK overexpressed-M610 cells (MF^-^9, MF^-^13) were cultured on dish for 24 hrs or in 0.1% collagen gel for 6 days. Assembled fibronectin was extracted and analyzed by Western blot using antibodies to detect fibronectin.

To determine whether FAK was involved in regulating tubule formation, all the FAK transfectants were cultured in 0.1% collagen gel for 6 days. FAK-overexpressed CA6 cells (CF5) continued to form cysts. The number and size of cysts were similar in CA6 cells and FAK-overexpressed CA6 cells (data not shown). In contrast, antisense FAK-overexpressed M610 cells (MF^-^9, MF^-^13) did not develop into tube-like structures, but instead formed small cell masses. In addition, decreasing the expression level of FAK in M610 cells reduced the tubule formation in a dose-dependent fashion (Fig. 6a). We next investigated whether FAK phosphorylation level was linked to tubule formation. FAK transfectants were cultured in 0.1% collagen gel for 6 days and FAK phosphorylation levels were examined. As shown in Fig. 6b, overexpression of FAK in CA6 cells did not augment FAKY397 phosphorylation. However, a decrease of FAK in M610 cells reduced FAKY397 phosphorylation. Taken together, our data indicate that FAK is required, but not sufficient for tubulogenesis.

Our previous study demonstrated that deposition of extracellular fibronectin is crucial for tubulogenesis [8]. To identify whether FAK-overexpressed CA6 cells fail to form tubules due to an inadequate fibronectin assembly, we analyzed the potential of fibronectin assembly in FAK transfectants cultured on dish for 24 hrs or in 0.1% collagen gel for 6 days. The results showed that overexpression of FAK in CA6 cells did not affect the amount of assembled fibronectin. In contrast, a decrease of FAK in M610 cells was accompanied by a reduction of fibronectin assembly (Fig. 6c). Thus, a decrease of FAK in M610 cells leads to down-regulation of tubule formation, which cannot be attributed to the reduction of fibronectin assembly. On the other hand, overexpression of wild type FAK in CA6 cells did not trigger tubulogenesis, which may be due to insufficient fibronectin assembly.

**Figure 7:**
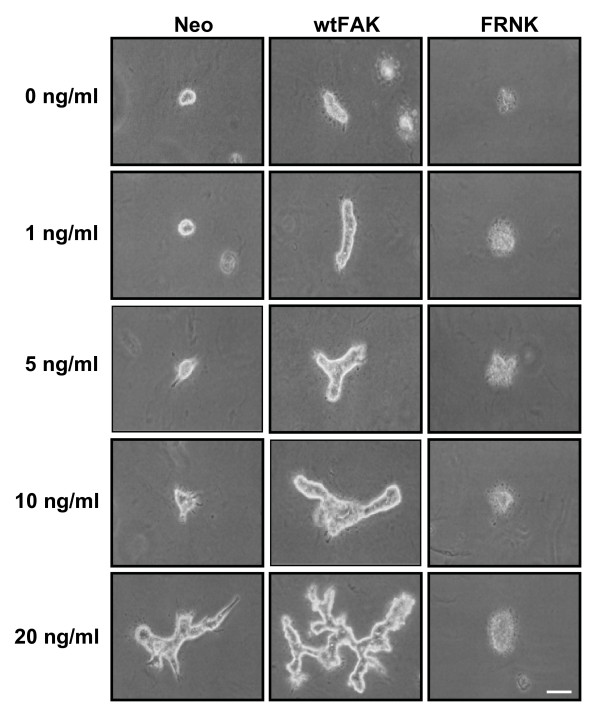
**The effect of FAK on tubulogenesis in 0.3% collagen gel with different dosage of hepatocyte growth factor**. Morphology of 3B5 cells stably transfected with FAK, FRNK, and Neo were cultured in 0.3% collagen gel in the presence of HGF of 0, 1, 5,10, 20 ng/ml, respectively, for 7 days. Bar = 100 μm.

To further investigate whether FAK is also involved in HGF-dependent tubulogenesis, 3B5 cells, a subclone of MDCK cells, harboring wild type FAK or dominant negative FRNK (FAK-related non-kinase) were employed. Cells were cultured in 0.3% collagen gel with different dosage regimen of HGF stimulation. FAK-overexpressed cells were more sensitive to HGF stimulation in forming branching tubules, whereas FRNK-overexpressed cells did not survive (Fig. 7). Taken together, our data show that FAK plays an important role in regulating branching tubulogenesis in both HGF-dependent and HGF-independent models.

## Discussion

In this study, we provide evidence that FAK is a key element in renal tubulogenesis. First, we isolated two different clones from MDCK cells, which proved to be a successful, HGF-independent model for tubule morphogenesis. We showed that mass clones forming tubule structures exhibited higher expression of FAK protein compared to cyst clones forming cystic morphogenesis in 0.1% collagen gel. Furthermore, the elevated FAK expression level in cyst clones cannot promote tubule formation. However, a decrease of FAK expression level in mass clones dramatically reduced branching tubule formation. Finally, we also demonstrated that FAK facilitates HGF-induced branching tubulogenesis since cells harboring wild type FAK sensitized the dose of HGF stimulation. These results indicate that FAK is involved in the regulation of both the HGF-dependent and HGF-independent branching morphogenesis.

The effects of FAK in kidney development *in vivo *have not been studied before, because knockout FAK in mice leads to embryonic lethal at day 8.5 [17]. However, observations from FAK-/- mice showed that FAK is an important regulator in vascular morphogenesis since FAK-deficient endothelial cell failed to organize into vascular networks [18,19]. Indeed, FAK has been shown to be required in blood vessel morphogenesis [20]. Furthermore, conditional FAK knockouts in endothelial cells led to defective angiogenesis [21]. Therefore, it is likely that conditional FAK knockouts in early ureteric bud may result in the failure of renal tubulogenesis *in vivo*.

Branching morphogenesis is a multi-step process that requires sequential cell adhesion to extracellular matrix, cell spreading, cell proliferation, and cell migration to ultimately form multicellular tube-like structures [22]. In the current study, we showed that decrease of FAK expression in M610 cells inhibited branching morphogenesis that was accompanied by the reduction of FAK phosphorylation and cell motility. However, overexpression of FAK in CA6 cells failed to elevate migration capacity, tubule formation and phosphorylation of FAK Y397 in collagen gel. Since there was a high agreement between the phosphorylation level of FAK Y397 and tubule formation, it is likely that FAK 397 phosphorylation may also play an important role in renal morphogenesis in MDCK cells.

Our previous study showed that MDCK cells cultured in collagen gel exhibited deposition of fibronectin underlying the epithelium, which may prevent apoptosis and therefore can possibly facilitate cyst or tubule growth of MDCK cells [7,8]. Recently, FAK was reported to promote organization of fibronectin matrix in endothelial cells [23]. Likewise, FAK may also regulate fibronectin assembly in MDCK cells. We reason that the decrease of FAK expression in M610 cells results in poor fibronectin deposition and hence leads to inhibition of branching tubulogenesis. Moreover, it has been reported that FAKY397 phosphorylation is highly regulated by substratum stiffness [24]. Well-assembled fibronectin around the cell may serve as a stiff substrate to further promote FAKY397 phosphorylation. Thus, mass clones are likely to possess higher potential to assemble fibronectin and to induce greater FAK phosphorylation. Finally, there may be a positive feedback loop in regulating FAK phosphorylation and fibronectin assembly during renal tubulogenesis.

To summarize, we have shown that FAK is critical for renal branching tubulogenesis through regulation of cell migration and fibronectin assembly. Our studies revealed FAK is involved in renal tubulogenesis, which may be also involved in other tissue morphogenesis such as mammary gland, pancreas or lung.

## Competing interests

The authors declare that they have no competing interests.

## Authors' contributions

WCW participated in the design and performed statistical analysis of the results and drafted the manuscript. AKK carried out part of the experimentation. MJT conceived of the study and participated in its design and coordination of the study. All authors read and approved the final manuscript.
